# Superiority of anthracycline‐free treatment in standard‐risk acute promyelocytic leukemia: A systematic review and comparative epidemiological analysis

**DOI:** 10.1002/cnr2.2035

**Published:** 2024-03-20

**Authors:** Kane Langdon, Stevie Cosentino, Olivia Wawryk

**Affiliations:** ^1^ College of Medicine and Dentistry James Cook University Cairns Queensland Australia; ^2^ Division of Medicine Cairns Base Hospital Cairns Queensland Australia; ^3^ Division of Medicine, Dentistry and Health Sciences University of Melbourne Melbourne Victoria Australia

**Keywords:** acute promyelocytic leukemia, all‐trans retinoic acid, arsenic trioxide, chemotherapy, epidemiology

## Abstract

**Background:**

Recent advances in the treatment of acute promyelocytic leukemia (APML) have seen unprecedented improvements in patient outcomes. However, such rapid growth in understanding often leads to uncertainty regarding superiority among candidate treatment regimens, especially when further scrutinized from an epidemiological perspective.

**Aims:**

The aim of this systematic review with epidemiological analysis was to identify and compare commonly utilized protocols for standard‐risk APML with a particular focus on complete remission (CR), overall/disease‐free survival (DFS), and reported adverse events.

**Methods and Results:**

Medline, Scopus, and CINAHL were interrogated to identify studies utilizing all‐trans retinoic acid (ATRA) in addition to arsenic trioxide (ATO) and/or anthracyclines such as idarubicin (IDA) in the treatment of de‐novo APML. After collation of studies, an epidemiological analysis was subsequently performed to compare protocols with regards to outcomes of interest using number needed to benefit (NNB) and number needed to harm (NNH) measures.

Seventeen articles, describing 12 distinct trials, were included in the analysis. These trials made use of three unique protocols; CR rates were 94%–100% for ATO/ATRA regimens, 95%–96% for ATO/ATRA/anthracycline regimens, and 89%–94% for ATRA/anthracycline regimens. Epidemiological analysis demonstrated NNB for CR was 9.09 (ATO/ATRA vs. ATRA/IDA) and 20.00 (ATO/ATRA vs. ATO/ATRA/IDA), NNH for neutropenia was −3.45 (ATO/ATRA vs. ATRA/IDA), and NNH for infection was −3.13 (ATO/ATRA vs. ATRA/IDA) and −1.89 (ATO/ATRA vs. ATO/ATRA/IDA).

**Conclusion:**

The ATO/ATRA regimen is superior to chemotherapy‐containing protocols at inducing remission and promoting survival in patients with APML. The regimen is better tolerated than the proposed alternatives with fewer adverse events. Future research opportunities include quantifying APML epidemiology and pursuing oral arsenic as an option for simplification of therapeutic protocols.

## 1 INTRODUCTION

Acute promyelocytic leukemia (APML) is a subtype of acute myeloid leukemia (AML) with a global incidence estimated up to 0.42 per 100 000 people.^1^ The disease is characterized by a translocation between chromosomes 15 and 17,^2^ resulting in the excessive production of the PML‐RARα protein.^3^ Before the 1970s, diagnosis with this condition was a death sentence with a median survival of less than 1 week.^4^ However, insightful research has uncovered the efficacy of all‐trans retinoic acid (ATRA)^5^ and arsenic trioxide (ATO)^6^ in APML treatment—leading to a much‐improved prognosis with 6‐year disease free survival rates as high as 96%.^7^ Studies demonstrating non‐inferiority of chemotherapy‐free alternatives in induction^8^, ^9^ and consolidation^10^ have informed a recent shift in clinical practice toward the omission of anthracycline in treatment regimens.^11^ However, these treatment decisions are complicated by the severity of the disease itself at presentation with much of the treatment based on risk stratification. Indeed, patients with white cell counts (WCCs) greater than 10 × 10^9^/L at diagnosis are classified as high risk and typically receive more intensive therapy.^12^

Despite these general considerations for approaching the management of APML, there remains uncertainty about the role for anthracyclines in the treatment of this hematological malignancy. This is reflected in differences between consensus protocols for the treatment of APML. Indeed, protocols such as those published by the American National Comprehensive Cancer Network (NCCN) for low‐risk APML advocate for chemotherapy‐free induction regimens, with anthracycline‐based therapy only utilized when ATO is contraindicated or unavailable.^13^ Conversely, other protocols such as those published by eviQ –the consensus‐based protocol resource for Australia– include the options of both ATO/ATRA/anthracycline and chemotherapy‐free protocols for patients with the same risk profile of disease.^14^, ^15^ Furthermore, much of the literature evaluating these different regimens focus primarily on survival and remission outcomes to the exclusion of adverse events, meaning less is known about deleterious outcomes accompanying each treatment – necessitating further elucidation.

Considering all this uncertainty, the following clinical question will be addressed: in patients with standard‐risk, de‐novo APML, how does ATO/ATRA induction and consolidation therapy compare with anthracycline‐based chemotherapeutic protocols in achieving complete remission, improving overall/disease‐free survival, and minimizing adverse effects. Additionally, the unique significance of this present study is its incorporation of an epidemiological analysis in addition to a standard outcome synthesis. This approach allows for a more comprehensive examination of the potential benefits and risks of each treatment regimen. To achieve this end, after a brief introduction to historical and contemporary treatment of APML, this review will utilize a systematic search of the literature to identify and discuss key studies that have influenced our treatment protocols utilizing ATRA and ATO; juxtaposing this with a comparative epidemiological analysis between ATRA/ATO treatment and traditional anthracycline regimens. Finally, we will discuss ongoing opportunities for research in the treatment of APML.

### 1.1 APML – Treatment progression and contemporary consensus protocols

**FIGURE 1:**
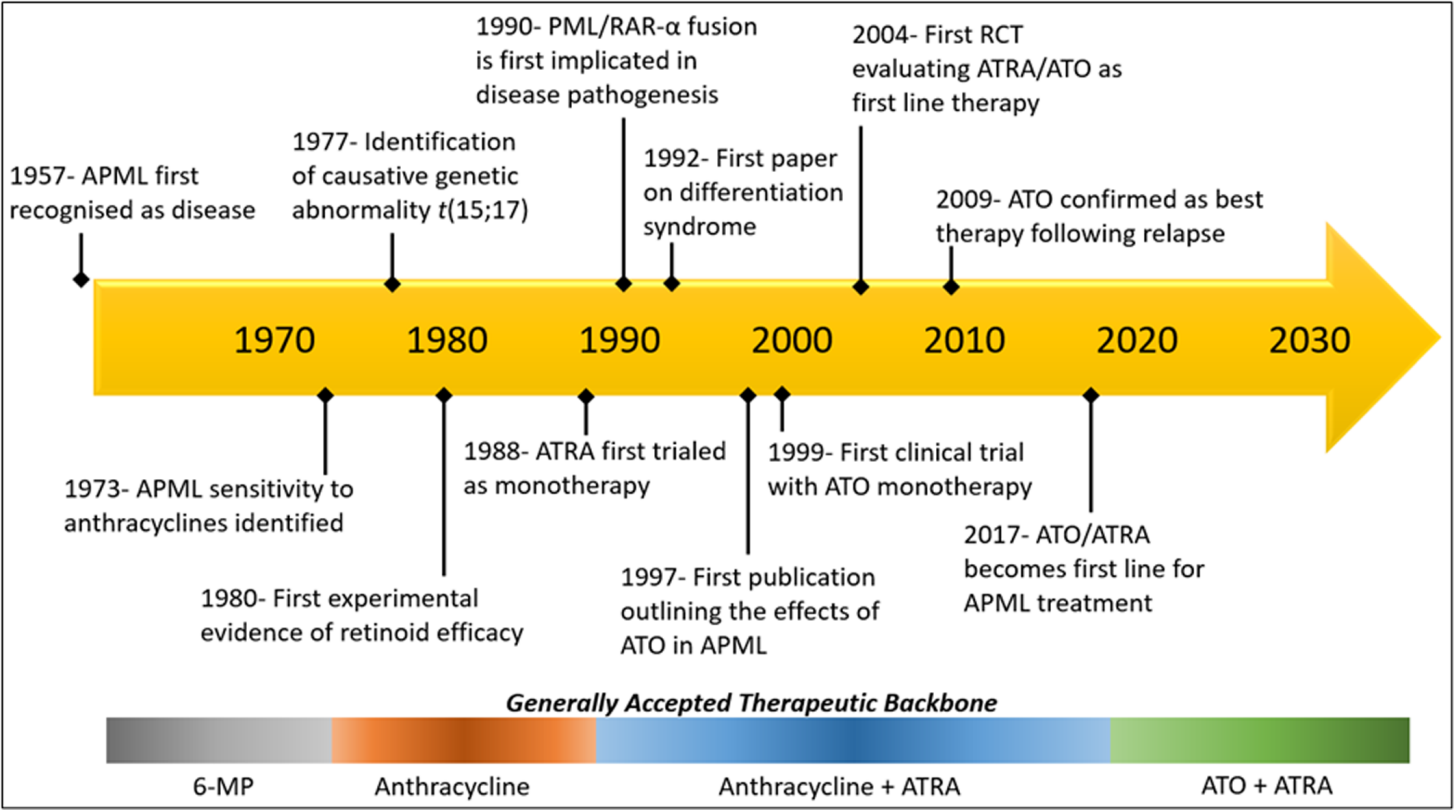
Timeline of significant events in the history of APML.

Many of the defining events in the history of APML treatment have been incorporated into Figure 1. First identified as a disease in its own right in 1957,^16^ APML was originally treated similarly to AML at the time with mercaptopurine‐based (6‐MP) regimes until it was discovered that anthracyclines had far superior efficacy in 1973.^17^ From there, significant research was undertaken into the genetic and molecular basis for the disease with the 15:17 translocation identified in 1977,^2^ and the subsequent PML/RARα fusion protein in 1990.^3^ During this time period, the use of retinoids began to be trialed as a way of inducing myeloid cellular differentiation with experimental evidence in 1980,^18^ and first clinical evidence as a monotherapy in 1988.^5^ The use of these vitamin A derivatives led to the identification of a potentially fatal syndrome beginning with fever and respiratory distress,^19^ now called differentiation syndrome.^20^ Thankfully, the syndrome is very amenable to treatment with dexamethasone.^19^ A more recent major advance in the history of APML is the discovery of the improved survival outcomes with the use of ATO. After the major effects of ATO on APML cells were described in 1997,^6^ numerous studies evaluated the effects of the drug with monotherapy initially assessed in 1999,^21^ and ATRA/ATO dual therapy first assessed in 2004.^22^ This explosion of studies led to ATO being confirmed as best treatment for relapsed disease in 2009,^23^ and an effective option for first‐line treatment in 2017^24^; causing protocols to be subsequently updated.

*eviQ* is an evidence‐based, consensus driven resource for Australian oncology treatment protocols, partnered with the government of New South Wales.^25^ After reviewing the evidence regarding different methods of treatment in APML, the organization has published two current protocols for the treatment of APML with minimal guidance for which to utilize in which context. One protocol utilizes a triple therapeutic backbone (ATO/ATRA/idarubicin [CHT Protocol]) and one utilizes a dual ATRA/ATO backbone (CHT‐Free Protocol). Additionally, the NCCN is an alliance of 33 different cancer centers around America which also provides evidence‐based, consensus guidelines for the treatment of a range of malignant conditions.^13^ While only stratifying patients into high‐ or low‐risk based on WCC at diagnosis (with WCC ≤10 × 10^9^/L classified as high‐risk),^13^ the NCCN recommends a regimen similar to *eviQ's* ATRA/ATO protocol (NCCN Protocol). The protocols for induction followed by a consolidation phase for management of APML recommended by these organizations have been summarized in Table 1.^14^, ^15^ As each of these have been recommended for low/standard‐risk APML, in this paper we will explore the benefits and adverse events associated with each protocol in order to provide suggestions for optimization of treatment outcomes for patients with the malignancy.

**TABLE 1:** Comparison of multiple consensus protocols for standard‐risk APML.^13^, ^14^, ^15^

Phase	NCCN protocol^13^	eviQ CHT protocol^14^	Eviq CHT‐free protocol^15^
Induction	Up to 60 days of: ATRA (45 mg/m^2^, daily, divided in two equal doses) ATO (0.15 mg/kg, daily) Weekly BMAT from day 28 until hCR or completion of cycle	36 days of: ATRA (45 mg/m^2^, daily, divided in two equal doses for days 1–36) IDA (12 mg/m^2^ on days 2, 4, 6, and 8) ATO (0.15 mg/kg, daily for days 9–36)	Up to 60 days of: ATRA (45 mg/m^2^, daily, divided in two equal doses) ATO (0.15 mg/kg, daily) Weekly BMAT from day 28 until hCR or completion of cycle
Consolidation	56 days/cycle for 4 cycles: ATRA (45 mg/m^2^, daily, for days 1–14 and 29–42^a^) ATO (0.15 mg/kg, daily for 5 days/week for 4 weeks from start of each cycle)	28 days of: ATRA (45 mg/m^2^, daily, divided in two equal doses) ATO (0.15 mg/kg, daily) *Followed in 3–4 weeks by*: 35 days of: ATRA (45 mg/m^2^, daily, divided in two equal doses for days 1–7, 15–21, and 29–35) ATO (0.15 mg/kg, daily for days 1–5, 8–12, 15–19, 22–26, and 29–33)	56 days/cycle for 4 cycles: ATRA (45 mg/m^2^, daily, divided in two equal doses for days 1–14 and 29–42^a^) ATO (0.15 mg/kg, daily for days 1–5, 8–12, 15–19, and 22–26)

Abbreviations: ATO, arsenic trioxide; ATRA, all‐trans retinoic acid; BMAT, bone marrow aspirate and trephine; CHT, chemotherapy; hCR, hematological complete remission; IDA, idarubicin.

^a^ Treatment on days 29–42 are not required in cycle 4 of 4.

## 2 METHODS

### 2.1 Literature search and inclusion/exclusion criteria

This review was prepared following the Preferred Reporting Items for Systematic Reviews and Meta‐analyses (PRISMA) statement where applicable (Appendix A).^26^ To answer the clinical question, a keyword and MeSH search strategy including Boolean operators was designed of the general form: *[APML] AND [ATO] AND [ATRA] AND [Clinical Trial]* (Appendix B). This strategy was utilized to identify original journal articles in Medline, Scopus, and CINAHL, searched from their inception to February 2023. The results were then screened against a pre‐determined list of inclusion and exclusion criteria.

#### 2.1.1 Inclusion

- English publications, describing a prospective clinical trial
- Standard‐risk, adult patients with de novo APML
- First‐line therapy or consolidation using ATRA with ATO and/or an anthracycline
- All years and geographical locations

#### 2.1.2 Exclusion

- Clinical trials where temporality of observations was not explicitly reported
- Patients post hematopoietic stem cell transplant
- Other hematological comorbidities
- Use of non‐anthracycline chemotherapy in consolidation regimens

Titles and abstracts were screened independently by two authors (KL and SC) who subsequently undertook separate full text reviews of the articles selected for potential inclusion. Differences in both screening and full text review stages were resolved by discussion. The reference lists of all included studies were screened for any further studies which fit the inclusion criteria by a single author (KL).

### 2.2 Data extraction and quality assessment

Data were extracted from text, figures, and/or tables of included studies by a single author (KL) with results then confirmed by a second author (SC). If the outcome measures were not reported in this form, the digital ruler tool from Nitro Pro 9^27^ was utilized to extract data from included graphs. If results differed between authors, the result in question was rechecked and a consensus was reached. Data extracted included number of participants in the study, age range, and Sanz risk score^12^; what treatment each study group received, complete remission (CR) percentage after induction, disease‐ or event‐free survival statistics (DFS/EFS), overall survival (OS) rates, and reported rates of grade 3–4 toxicities using the Common Terminology Criteria for Adverse Events scale.^28^ The time period for DFS, EFS and OS data was extracted exactly as reported in the literature with no interpolation performed. Studies were ordered chronologically with evidence synthesis carried out per treatment regimen. Where studies assessed multiple treatment regimens with only some meeting inclusion/exclusion criteria, characteristics of the entire study were extracted and included in the summary table, but the excluded regimens were not included in the textual qualitative analysis. Outcome measures were extracted for all time points with missing data excluded from synthesis. Study quality was assessed using a tool adapted from the McMaster Critical Review Form – Quantitative Studies.^29^ The analysis consisted of 20 criteria by which each paper was assessed, with the presence of each criterion being worth one point for a total of 20 points. The criteria were: purpose stated clearly [1], relevant literature reviewed [2], design appropriate to question [3], no biases present [4], sample described in detail [5], sample size justified [6], ethics approval reported [7], informed consent gained [8], outcome measures reliable [9], outcome measures valid [10], intervention described in detail [11], contamination avoided [12], cointervention avoided [13], statistical reporting of results [14], appropriate statistical analysis [15], clinical importance reported [16], drop‐outs reported [17], appropriate conclusions [18], clinical implications reported [19], study limitations acknowledged [20]. Studies were rated as poor (<7 points), moderate (7–11 points), good (12–15 points), very good (16–18 points), or excellent quality (19–20 points).

### 2.3 Comparative epidemiological analysis

To explore the benefit of ATO/ATRA therapy compared to the commonly accepted alternatives (ATO/ATRA/IDA and ATRA/IDA), a comparative epidemiological analysis was performed with focus on CR, OS, DFS/EFS, and adverse events during induction therapy. It was predetermined that the landmark studies for each of the key therapeutic regimens would be utilized for this analysis as they provided much of the initial evidence base for the treatment protocols. For the comparison of CR, OS, and DFS numbers needed to benefit (based on the numbers needed to treat statistic) was calculated using the formula^30^:

$$\mathrm{NNB}=\frac{1}{I_{e}-I_{u}}$$

where:

- *I*_*e*_ is the incidence of the outcome of interest in the treatment group, and
- *I*_*u*_ is the incidence of the same outcome in the control group.

Conversely, to compare adverse events between treatment regimens the number needed to harm statistic was calculated. Indeed, this statistic is virtually the same as NNB except the outcome in question is negative, therefore, it was calculated using the formula^30^:

$$\mathrm{NNH}=\frac{1}{I_{e}-I_{u}}$$

where:

- *I*_*e*_ is the incidence of the adverse event in the treatment group, and
- *I*_*u*_ is the incidence of the same event in the control group.

Where relevant in the analysis, statistical results were reported to two significant figures.

## 3 RESULTS

### 3.1 Search strategy

**FIGURE 2:**
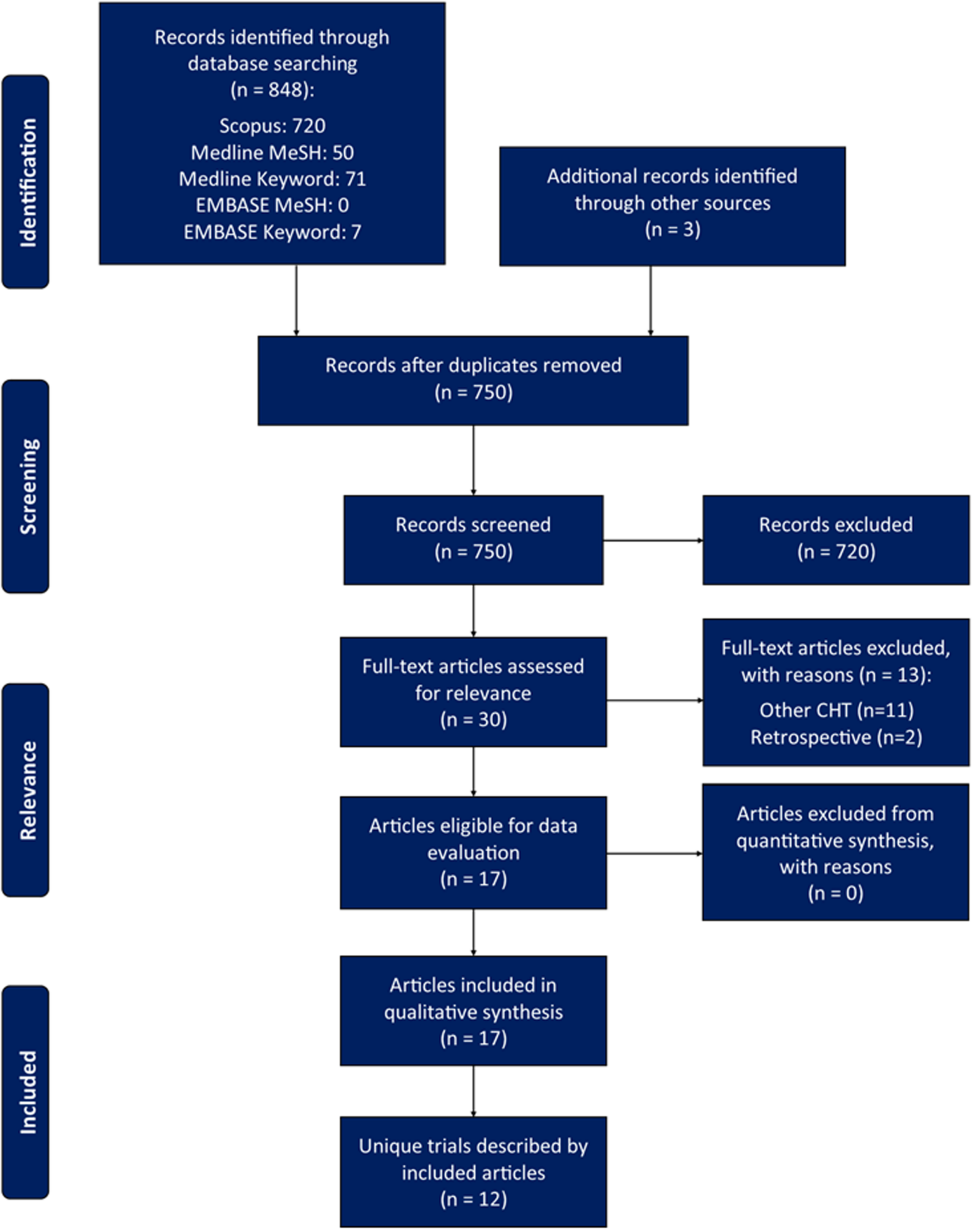
PRISMA flow diagram for selection of included studies.

The results of the search strategy have been compiled into Figure 2. Performing this search strategy resulted in the identification of a total of 848 articles; 720 from Scopus, 121 from MEDLINE, 7 from EMBASE and 3 from the reference list of included studies. After duplicates were removed, 750 articles were screened using title and abstract to identify any clinical trial evaluating the use of ATRA and arsenic and/or anthracyclines in the context of APML. The resulting 30 articles were assessed in full, with 13 articles excluded (including all 3 studies identified in reference list searching) due to use of other chemotherapeutics (*n* = 11) and the retrospective nature of the study (*n* = 2). Subsequently, 17 articles—describing 12 distinct trials—were identified and included in the qualitative synthesis.^7^, ^8^, ^9^, ^10^, ^24^, ^31^, ^32^, ^33^, ^34^, ^35^, ^36^, ^37^, ^38^, ^39^, ^40^, ^41^, ^42^

### 3.2 Study characteristics and quality

The key information from each of the 12 identified trials has been collated into Table 2. Studies were identified as early as 2006,^37^, ^38^ and as recently as 2022.^8^, ^9^ The majority of studies were performed by international collaborative research groups with study sizes up to 581 patients.^33^ Patient age groups were heterogenous in these studies but typically included patients between the ages of 18 and 70. Five studies focused on low to intermediate‐risk patients,^7^, ^24^, ^33^, ^35^, ^37^, ^38^, ^40^ with the remaining seven including all risk categories.^8^, ^9^, ^10^, ^31^, ^32^, ^34^, ^36^, ^39^, ^41^, ^42^ Regarding induction treatment, five papers evaluated an ATRA/anthracycline regimen with CR rates from 89%–94%,^32^, ^34^, ^36^, ^37^, ^38^, ^39^, ^41^ two studies evaluated an ATO/ATRA/anthracycline regimen with CR rates from 95%–96%,^10^, ^31^, ^42^ and five studies evaluated an ATO/ATRA regimen with CR rates from 94%–100%.^7^, ^8^, ^9^, ^24^, ^32^, ^34^, ^35^, ^40^ Consolidation treatment with ATRA/anthracyclines was assessed in six studies with DFS from 77% (2‐year) to 93% (7‐year),^10^, ^32^, ^33^, ^34^, ^36^, ^37^, ^38^, ^39^ ATO/ATRA/anthracyclines was assessed in one study with DFS of 98% (2‐year),^31^, ^42^ and ATO/ATRA was assessed in six studies with DFS from 91% (4‐year) to 96% (7‐year).^7^, ^8^, ^9^, ^10^, ^24^, ^32^, ^34^, ^35^, ^40^

**TABLE 2:** Clinical trials evaluating ATO/ATRA in the treatment of APML.

Trial/Author (Year)^Ref^	Study group or location	*n*	Age	Risk group	Treatment arms	CR/OS statistics	Grade 3–4 toxicities	Key findings/policy changes	Quality
APL2000 (2006)^37^, ^38^	European APL group	340	30–51	L + I	ATRA + DNR versus ATRA + DNR + ARA‐C	CR: 94% versus 99% 2‐year DFS/OS: 77% versus 93%/ 90% versus 98%	NA.	Initial recommendation for inclusion of ARA‐C in DNR induction and consolidation regimens.	Very good
Aljurf (2010)^36^	Saudi Arabia	25	13–78	All	ATRA + IDA	CR: 92% 3.6‐year DFS: 78% 4.2‐year OS: 90%	Nil.	ATRA + IDA induction followed by simplified post‐remission ATRA + IDA maintains satisfactory DFS.	Very good
APML3 (2012)^39^	ALLG	101	19–73	All	ATRA + IDA	CR: 90% 4‐year DFS: 70% 4‐year OS: 84%	NA.	ATRA + IDA confers prolonged DFS in majority of cases.	Very good
APML4^a^ ^,^ ^b^ (2012)^31^, ^42^	ALLG	123	3–78	All	ATO + ATRA + IDA versus historic control (APML3 above)	CR: 95% 2‐year DFS: 98% 2‐year OS: 93%	Cardiac: 1% GI: 28% Infection: 76% Hepatic 44% QTc prolonged: 14%	ATRA + IDA + ATO conferred significantly better disease‐free survival than ATRA + IDA above.	Very good
Lo‐Coco^b^ (2013)^40^	GIMEMA, AMLSG, SAL	162	18–71	L + I	ATO + ATRA versus ATRA + IDA + MTZ	CR: 100% versus 95% 2‐year EFS/OS: 97% versus 85%/ 99% versus 91%	Neuts: 46% versus 79% PLT: 59% versus 88% Hepatic: 63% versus 6% QTc prolonged: 16% versus 0%	ATO + ATRA non‐inferior to ATRA + anthracycline approach in low/intermediate risk APML.	Excellent
SAL‐AIDA2000 (2014)^41^	Germany	141	19–82	All	ATRA + IDA ➔ 3 cycles versus 2 cycles of consolidation with ATRA + MTZ + IDA.	CR: 92% 3‐year OS: 82%	NA.	2 cycles of consolidation in chemotherapeutic protocol non‐inferior to traditional 3 cycles.	Very good
AML17^a^ (2015)^32^, ^34^	NCRI AMLWG	235	16–77	All	ATO + ATRA versus ATRA + IDA	CR: 94% versus 89% 2‐year EFS/OS: 98% versus 91%/ 94% versus 89% 4‐year EFS/OS: 91% versus 70%/ 97% versus 78%	Cardiac: 2% versus 6% GI: 1% versus 10% Hepatic: 29% versus 14%	ATRA + ATO non‐inferior overall survival and superior relapse‐free survival rates.	Very good
APL0406^a^ ^,^ ^b^ (2017)^7^, ^24^	GIMEMA, AMLSG, SAL	276	18–71	L + I	ATO + ATRA versus ATRA + IDA + MTZ	CR: 100% versus 97% 50‐month EFS/OS: 97% versus 80%/ 99% versus 93%	Neuts: 35% versus 64% PLT: 38% versus 62% Infection: 23% versus 55% Hepatic: 40% versus 3% QTc prolonged: 9% versus 1%	Continuation of Lo‐Coco study to meet QoL endpoint. Superior survival causes recommended treatment to become ATRA + ATO for mild‐moderate APML.	Very good
APL 2006 (2018)^33^	French‐Belgian‐Swiss APL group	581	</=70	L + I	ATRA + IDA + ARA‐C ➔ consolidation with ARA‐C + IDA versus ATO + IDA versus ATRA + IDA	CR: 98% 5‐year EFS: 89% versus 96% versus 85%	NA.	Recommended consolidation regime to include ATO.	Very good
Kayser (2021)^35^	Germany, France	154	>18	L + I	Single arm ATO + ATRA	CR: 99% 2‐year OS: 95%	Hematologic: 4% Hepatic: 18% QTc prolonged: 2%	Confirmation of efficacy/durability of ATO + ATRA as initial therapy in low + intermediate risk.	Very good
APL2012 (2021)^10^	China	855	18–65	All	ATO + ATRA + IDA/DNR ➔ consolidation with ATO + ATRA versus ATRA + IDA/DNR ± ARA‐C	CR: 96% 7‐year DFS/OS: 96% versus 93%/97% versus 97%	Neuts: 56% versus 96% PLT: 15% versus 72% Hepatic: 0.3% versus 0.5% QTC prolonged: 4% versus 0.4%	Non‐inferiority of ATO + ATRA consolidation with favorable adverse effect profile.	Excellent
APL15 (2022)^8^, ^9^	China	128	15–80	All	ATO + ATRA versus ATO + ATRA + IDA + ARA‐C	CR: 97% versus 97% 2‐year DFS: 98% versus 97%	Neuts: 8% versus 17% PLT: 32% versus 45% Hepatic: 8% versus 8% Renal: 2% versus 0% Cardiac: 2% versus 12% QTC prolonged: 0% versus 2%	Non‐inferiority of ATO/ATRA as initial treatment in all risk groups.	Very good

Abbreviations: ARA‐C, cytarabine; DNR, daunorubicin; GI, gastrointestinal; IDA, idarubicin; L + I, low and intermediate risk; MTZ, mitoxantrone; Neuts, neutropenia; PLT, thrombocytopaenia; QoL, quality of life.

^a^ Indicates a paper of particular importance included in the epidemiological analysis.

^b^ Indicates a paper referenced by eviQ guidelines.

Grade 3–4 treatment toxicity was reported in seven studies.^7^, ^8^, ^9^, ^10^, ^24^, ^31^, ^32^, ^34^, ^35^, ^40^, ^42^ Typically, anthracycline‐based regimens were more likely to cause hematological or infection‐related adverse events (up to 96%^10^ and 55%^7^, ^24^ of patients, respectively). Conversely, regimens containing ATO were more likely to cause liver injury and QTc prolongation (up to 63%^40^ and 16%^40^ of patients respectively). Overall, all included studies were of high quality, with scores ranging from very good to excellent quality. Most commonly, studies lost points due to the risk of bias, lack of justification of sample size, or not acknowledging limitations. The full assessment of the quality of included studies can be found in Appendix C.

### 3.3 Comparative epidemiology

Identified landmark studies in the development of current APML treatment protocols were the APML4 study for ATO/ATRA/IDA treatment,^31^, ^42^ APL0406 (the subsequent expansion and further analysis of the Lo‐Coco et al. cohort) for ATO/ATRA treatment,^7^, ^24^, ^40^ and AML17 for ATRA/IDA treatment (given this study was the primary non‐inferiority study comparing the two common regimens).^32^, ^34^ As determined a priori, these studies were therefore utilized for the comparative epidemiological analysis. The results of the analysis are presented in Table 3.

**TABLE 3:** Epidemiological analysis for the use of ATO/ATRA when compared to ATO/IDA and ATO/ATRA/IDA.

Outcome of interest	ATO/ATRA versus ATRA/IDA	ATO/ATRA versus ATO/ATRA/IDA
*Number needed to benefit*		
Complete remission (CR)	9.09	20.00
2‐year survival (2‐YS)	10.00	16.67
4‐year survival	4.76	−^b^
4‐year event free survival	3.70	−^b^
*Number needed to harm (during induction)*		
Grade 3–4 neutropenia	−3.45^a^	−^b^
Grade 3–4 thrombocytopenia	−4.17^a^	−^b^
Infection	−3.13^a^	−1.89
QTc prolongation	12.50^a^	−20.00
Grade 3–4 liver injury	3.85	−25.00

^a^ Indicates the statistic was taken from APL0406,^7^, ^24^ as it was not reported in AML17.^32^, ^34^

^b^ Indicates that this data point was not reported in the APML4 trial.^31^

Comparing ATO/ATRA therapy to ATRA/IDA, the NNB for CR was 9.09 patients and 4‐year OS and EFS was 4.76 and 3.70 patients respectively. Regarding adverse events, NNH for neutropenia and thrombocytopenia were −3.45 and −4.17 patients respectively. NNH for infection was −3.13 patients. However, NNH for QTc prolongation was 12.82 patients and liver injury was 3.85 patients.

When comparing ATO/ATRA therapy to the ATO/ATRA/IDA regimen, the NNB for CR and 2‐year survival were 20.00 and 16.67 patients respectively. Unfortunately, adverse event outcome data was only reported for infection and QTc prolongation in the APML4 study.^31^, ^42^ Nonetheless, NNH for infection was −1.89 patients, for QTc prolongation was −20.00 patients, and for liver injury was −25.00 patients.

## 4 DISCUSSION

The primary objective of this systematic review was to evaluate the contemporary ATO/ATRA therapeutic approach against the historical anthracycline‐containing regimens. It is evident that this chemotherapy‐free protocol is superior to historical regimens (ATRA/IDA and ATO/ATRA/IDA) in terms of both inducing CR and maintaining DFS. The main reason for patients not reaching CR in the clinical trial literature is early death.^43^ Studies have typically attributed this terrible outcome to delayed time to diagnosis/specialty treatment, inadequate supportive care, and severe infection or hemorrhage.^44^ Interestingly, our study has highlighted improved CR rates can be found using the chemotherapy‐free regimen with reductions also identified in grade 3–4 neutropenia, thrombocytopenia and infection when compared to both chemotherapy‐containing regimens. As such, a potential additional risk factor for early death (and subsequently reduced CR rates) in patients receiving anthracycline‐based regimens may be adverse events secondary to the aggressive use of chemotherapeutics.

Consolidation regimens and adverse event rates further demonstrate the superiority of the ATO/ATRA protocol. DFS was clearly higher in the ATO/ATRA regimens when compared to those utilizing ATRA/IDA. However, there was only one study which evaluated ATO/ATRA/IDA consolidation which only provided DFS and OS data to 2 years. As such, it is difficult to make comparative statements between DFS and OS in ATO/ATRA versus ATO/ATRA/IDA protocols. Regarding adverse events, there were favorable rates of hematological, gastrointestinal and infection adverse events in the chemotherapy‐free protocol. However, inclusion of ATO into the therapeutic regimen was associated with increased risk of liver injury and QTc prolongation—known side effects of ATO use.^45^, ^46^ Fortunately, significant arrythmias from ATO‐induced QTc prolongation seem to be rare.^46^ Nonetheless, these adverse events commonly affect appropriateness of contemporary treatment regimens in patients with significant comorbidities such as pre‐existing liver dysfunction or QTc prolongation.^47^

The benefit of utilizing chemotherapy‐free regimens is further demonstrated in the comparative epidemiological analysis. The NNB/NNH statistics are a measure of how many patients need to be treated with ATRA/ATO rather than the comparator for a single patient to experience an additional benefit (for NNB) or harm (for NNH). A negative result indicates the event in question is less likely to occur. As such, it is important to note that only nine patients need be treated with ATO/ATRA rather than ATO/IDA for an additional patient to reach CR. As the most common cause for not reaching CR in our included studies is early death, this statistic becomes considerably more clinically significant. Of further note is that approximately four patients need be treated with ATO/ATRA rather than ATO/IDA for an additional patient to reach 4 years post diagnosis without relapse of APML. While the NNB for ATO/ATRA versus ATO/ATRA/IDA are slightly higher, there are consistent improvements in epidemiological outcomes for both CR and OS. From an NNH perspective, only three to four patients need be treated with ATO/ATRA rather than ATRA/IDA for a single patient to not experience an episode of neutropenia, thrombocytopenia, or infection. Of additional note, for every 13 and four patients treated with ATO/ATRA rather than ATRA/IDA, one additional patient does experience QTc prolongation and liver injury respectively. Finally, when assessing the reported data points comparing adverse events between ATO/ATRA and ATO/ATRA/IDA, it is evident that the chemotherapy‐free protocol is associated with favorable outcomes throughout, with a particular reduction in the rates of infections. As such, from a long‐term health‐related quality of life perspective, treatment with the ATO/ATRA regimen is more likely to result in an improved outlook when compared to the ATO/ATRA/IDA and ATRA/IDA regimens.

Traditionally, APML patients considered to be high‐risk (white cell count >10 × 10^9^/L at diagnosis via the Sanz score)^12^ were treated with some form of chemotherapy in an attempt to reduce white cell counts and minimize risk of differentiation syndrome and poorer outcomes due to the pro‐differentiation effect of ATRA.^48^ Interestingly, the recent APL2012^10^ and APL15^8^, ^9^ trials have provided contemporary evidence for non‐inferiority of the chemotherapy‐free protocol in both consolidation and induction for all‐risk patients—albeit with the initial use of gemtuzumab ozogamicin or hydroxyurea to control for hyperleukocytosis.^8^, ^9^, ^10^ These findings have been further supported by independent, real‐world data including from Indian centers emphasizing abbreviated courses of anthracycline,^49^ or high‐dose hydroxyurea.^50^ As such, with the identified reduced adverse event profile of the chemotherapy‐free protocol it is likely that these new findings will cause a shift in treatment paradigm toward ATO/ATRA for high‐risk patients—similar to the chemotherapy‐free treatment shift in standard‐risk patients witnessed after publication of the Lo‐Coco et al. and APL0406 trials.^7^, ^24^, ^40^

Regarding the included papers as a whole, a major strength is that the majority these studies were of very good quality from international, multi‐center, randomized controlled trials—constituting level II evidence on the National Health and Medical Research Council evidence hierarchy.^51^ Indeed, much of the study space in APML is dominated by international collaborative efforts between study groups such as the Australasian Leukemia & Lymphoma Group (ALLG),^31^, ^39^, ^42^ the Gruppo Italiano Malattie EMatologiche dell'Adulto [the Italian Adult Hematological Diseases Group] (GIMEMA), German‐Austrian Acute Myeloid Leukemia Study Group (AMLSG) and Study Alliance Leukemia,^7^, ^24^, ^40^ and the National Cancer Research Institute Acute Myeloid Leukemia Working Group (NCRI AMLWG).^32^, ^34^ However, studies were consistently at risk of introducing biases. Studies evaluating only low/intermediate‐risk patients were naturally introducing selection bias, however, this was appropriately reflected in most discussions of clinical implications with caution warned for generalizability to full APML cohorts. Additionally, APML has been traditionally associated with a high numbers of early death, quoting rates of 5%–10% of patients in the trial literature^39^ and up to 29% in population studies.^52^ As such, it is inevitable that clinical trials on this population have some degree of selection bias—irrespective of which risk groups are included. As induction therapy becomes optimized early death seems to be reducing, with CR rates of 97%–99% for patients receiving ATO/ATRA in studies published over the last 5 years.^8^, ^9^, ^35^ As such, selection bias is likely to be inversely proportionate to the improving rates of CR. Furthermore, while it was common for patient dropouts to be reported and intention to treat analyses to be performed, this was not standard across all studies. Hence, risk of attrition bias was also commonly introduced. Finally, considering the cardiac risk profile associated with the use of ATO, especially regarding patients diagnosed with congestive heart failure or prolonged QTc,^47^ the findings of this study should be used in caution when considering these specific patient populations.

This review itself also has key strengths and limitations. The main strength of this study is that it was performed in a systematic manner with the inclusion/exclusion criteria and planned evidence synthesis determined a priori, minimizing risk of bias introduced through the subsequent analysis. Furthermore, the review was carried out in accordance with PRISMA guidelines where applicable,^26^ and an adapted quality appraisal tool was utilized. Conversely, the limitations of this study include that paper selection and extraction was performed by a single author, the planned inclusion/exclusion criteria and data synthesis was not published as a protocol prior to data extraction, and the evidence synthesis was not as robust as a complete meta‐analysis with the calculation of a standardized effect size.

### 4.1 Future opportunities in APML – Research and treatment

From a therapeutic perspective, opportunities center around confirming and optimizing current protocols. While the APL15 trial has provided initial evidence for non‐inferiority of chemotherapy‐free treatment in all‐risk APML,^8^, ^9^ additional randomized controlled trials are required to confirm these findings—especially in dissimilar population groups. Furthermore, an oral arsenic formulation has been commercially available for APML treatment in Chinese populations since 2009 with studies demonstrating non‐inferiority of the formulation compared to intravenous ATO.^53^ The oral formulation has the benefit of being able to be administered outside of hospital, with subsequently reduced administration costs.^53^ Drawing on earlier studies such as the American phase 1 clinical trial by Ravandi et al. which demonstrated safety and bioavailability of oral arsenic in advanced hematological disease,^54^ a similar phase 1 study is currently being conducted specifically in APML by the ALLG in Australia and New Zealand.^55^ If the trial is successful, the next opportunity will be for phase 2/3 clinical trials further evaluating the use of oral arsenic in APML.

From an epidemiological perspective, there are numerous research opportunities in delineating APML data to assist in further guiding research direction. As previously mentioned, CR statistics of included studies suggest that early death rates have reduced with optimization of therapies which is reflected in a recent population study.^56^ However, there is further opportunity in confirming these findings, particularly with regards to risk factors and frequency of early intracranial death during induction. This could be performed in other large cohorts, both within subpopulations of pre‐existing American groups and elsewhere around the world. Indeed, Oceania particularly seems to have a paucity of data with Australian‐specific statistics for both APML and acute myeloid leukemia generally lacking. Only a single study by Gangatharan et al. presenting data from 2005 has addressed Australian‐based APML statistics.^57^ Furthermore, the most recent study evaluating epidemiology of AML only presented data as recently as 2016.^58^ Much of the treatment landscape has evidently changed in this time, especially since the 2005 data. As such, it is imperative that further data at a global level is collected from both an AML and APML perspective.

## 5 CONCLUSION

Our understanding of the optimal treatment for APML has significantly advanced since the turn of the century with a number of major developments. In this paper, our research question was in patients with standard‐risk, de novo APML, how does ATO/ATRA induction and consolidation therapy compare with anthracycline‐based chemotherapeutic protocols in achieving complete remission, improving overall/disease‐free survival, and minimizing adverse effects. Our review has demonstrated that the use of ATO with ATRA in the treatment of APML is superior to chemotherapy‐containing protocols at inducing remission and promoting survival in patients with APML. The regimen is better tolerated than the proposed alternatives with fewer adverse events, excepting those relating to QTc prolongation and liver injury, which is reflected in our epidemiological analysis. Hence, the significance of this study is its synthesis of traditional outcome measures from extensive clinical trials with the addition of a patient‐focused epidemiological perspective to highlight and explore optimal treatment strategies for APML. The results are applicable to all patients with the disease, especially those classified as standard‐risk, and the hematologists by whom they are treated. Indeed, due to contemporary treatment protocols, the diagnosis of APML has been transformed from the equivalent of a death sentence to one of the most treatable hematological malignancies. In the future, major research opportunities are to further quantify the epidemiological statistics of APML given recent paradigm shifts in treatment and investigate the option of oral arsenic in streamlining outpatient management.

## AUTHOR CONTRIBUTIONS

**Kane Langdon:** Conceptualization (equal); data curation (equal); formal analysis (equal); investigation (equal); methodology (equal); project administration (equal); software (equal); validation (equal); visualization (equal); writing – original draft (equal); writing – review and editing (equal). **Stevie Cosentino:** Data curation (equal); formal analysis (equal); investigation (equal); methodology (equal); project administration (equal); validation (equal); writing – original draft (equal); writing – review and editing (equal). **Olivia Wawryk:** Data curation (equal); formal analysis (equal); investigation (equal); methodology (equal); project administration (equal); supervision (lead); validation (equal); writing – original draft (equal); writing – review and editing (equal).

## CONFLICT OF INTEREST STATEMENT

The authors have stated explicitly that there are no conflicts of interest in connection with this article.

## ETHICS STATEMENT

This study did not require ethics approval due to its systematic review design.

## Data Availability

The data that support the findings of this study are available from the corresponding author upon reasonable request.

## APPENDIX A PRISMA CHECKLIST

### A.1

**Table:**

Section and topic	Item #	Checklist item	Page where item is reported
**TITLE**			
Title	1	Identify the report as a systematic review.	1
**ABSTRACT**			
Abstract	2	See the PRISMA 2020 for Abstracts checklist.	N/A
**INTRODUCTION**			
Rationale	3	Describe the rationale for the review in the context of existing knowledge.	3–7
Objectives	4	Provide an explicit statement of the objective(s) or question(s) the review addresses.	4–5
**METHODS**			
Eligibility criteria	5	Specify the inclusion and exclusion criteria for the review and how studies were grouped for the syntheses.	7–9
Information sources	6	Specify all databases, registers, websites, organisations, reference lists and other sources searched or consulted to identify studies. Specify the date when each source was last searched or consulted.	7
Search strategy	7	Present the full search strategies for all databases, registers and websites, including any filters and limits used.	7, 34
Selection process	8	Specify the methods used to decide whether a study met the inclusion criteria of the review, including how many reviewers screened each record and each report retrieved, whether they worked independently, and if applicable, details of automation tools used in the process.	7–8
Data collection process	9	Specify the methods used to collect data from reports, including how many reviewers collected data from each report, whether they worked independently, any processes for obtaining or confirming data from study investigators, and if applicable, details of automation tools used in the process.	8
Data items	10a	List and define all outcomes for which data were sought. Specify whether all results that were compatible with each outcome domain in each study were sought (e.g. for all measures, time points, analyses), and if not, the methods used to decide which results to collect.	8–9
	10b	List and define all other variables for which data were sought (e.g. participant and intervention characteristics, funding sources). Describe any assumptions made about any missing or unclear information.	8
Study risk of bias assessment	11	Specify the methods used to assess risk of bias in the included studies, including details of the tool(s) used, how many reviewers assessed each study and whether they worked independently, and if applicable, details of automation tools used in the process.	8–9
Effect measures	12	Specify for each outcome the effect measure(s) (e.g. risk ratio, mean difference) used in the synthesis or presentation of results.	8–9
Synthesis methods	13a	Describe the processes used to decide which studies were eligible for each synthesis (e.g. tabulating the study intervention characteristics and comparing against the planned groups for each synthesis (item #5)).	8–9
	13b	Describe any methods required to prepare the data for presentation or synthesis, such as handling of missing summary statistics, or data conversions.	9–10
	13c	Describe any methods used to tabulate or visually display results of individual studies and syntheses.	9
	13d	Describe any methods used to synthesize results and provide a rationale for the choice(s). If meta‐analysis was performed, describe the model(s), method(s) to identify the presence and extent of statistical heterogeneity, and software package(s) used.	N/A
	13e	Describe any methods used to explore possible causes of heterogeneity among study results (e.g. subgroup analysis, meta‐regression).	N/A
	13f	Describe any sensitivity analyses conducted to assess robustness of the synthesized results.	N/A
Reporting bias assessment	14	Describe any methods used to assess risk of bias due to missing results in a synthesis (arising from reporting biases).	N/A
Certainty assessment	15	Describe any methods used to assess certainty (or confidence) in the body of evidence for an outcome.	N/A
**RESULTS**			
Study selection	16a	Describe the results of the search and selection process, from the number of records identified in the search to the number of studies included in the review, ideally using a flow diagram.	10–11, 23
	16b	Cite studies that might appear to meet the inclusion criteria, but which were excluded, and explain why they were excluded.	10–11
Study characteristics	17	Cite each included study and present its characteristics.	10–12, 29–30
Risk of bias in studies	18	Present assessments of risk of bias for each included study.	29–30, 35
Results of individual studies	19	For all outcomes, present, for each study: (a) summary statistics for each group (where appropriate) and (b) an effect estimate and its precision (e.g. confidence/credible interval), ideally using structured tables or plots.	29–30
Results of syntheses	20a	For each synthesis, briefly summarise the characteristics and risk of bias among contributing studies.	11–12, 29–30
	20b	Present results of all statistical syntheses conducted. If meta‐analysis was done, present for each the summary estimate and its precision (e.g. confidence/credible interval) and measures of statistical heterogeneity. If comparing groups, describe the direction of the effect.	11–12, 31
	20c	Present results of all investigations of possible causes of heterogeneity among study results.	N/A
	20d	Present results of all sensitivity analyses conducted to assess the robustness of the synthesized results.	N/A
Reporting biases	21	Present assessments of risk of bias due to missing results (arising from reporting biases) for each synthesis assessed.	N/A
Certainty of evidence	22	Present assessments of certainty (or confidence) in the body of evidence for each outcome assessed.	N/A
**DISCUSSION**			
Discussion	23a	Provide a general interpretation of the results in the context of other evidence.	13–15
	23b	Discuss any limitations of the evidence included in the review.	15–16
	23c	Discuss any limitations of the review processes used.	17
	23d	Discuss implications of the results for practice, policy, and future research.	17–18
**OTHER INFORMATION**			
Registration and protocol	24a	Provide registration information for the review, including register name and registration number, or state that the review was not registered.	17
	24b	Indicate where the review protocol can be accessed, or state that a protocol was not prepared.	17
	24c	Describe and explain any amendments to information provided at registration or in the protocol.	N/A
Support	25	Describe sources of financial or non‐financial support for the review, and the role of the funders or sponsors in the review.	1
Competing interests	26	Declare any competing interests of review authors.	1
Availability of data, code and other materials	27	Report which of the following are publicly available and where they can be found: template data collection forms; data extracted from included studies; data used for all analyses; analytic code; any other materials used in the review.	1

## APPENDIX B SEARCH STRATEGY

### B.1

***MeSH Search* (performed in all databases where MeSH terms were applicable)**

exp/[Leukemia, Promyelocytic, Acute] AND exp/[Arsenic Trioxide] AND exp/[Tretinoin] AND exp/[Clinical Trial]

***Keyword Search* (performed in all databases irrespective of option for use of MeSH terms)**

(APML OR “aml m3” OR “m3 aml” OR “anll m3” OR “m3 anll” OR “acute promyelocytic leukemia” OR “acute promyelocytic leukemia” OR “acute promyelocytic leukemias” OR “acute promyelocytic leukemias” OR “progranulocytic leukemia” OR “progranulocytic leukemia”)

AND

(“1327‐53‐3” OR “arsenic oxide” OR “arsenic trioxide” OR arsenolite OR “arsenous anhydride” OR as2o3 OR as4o6 OR “diarsenic trioxide” OR naonobin OR s7v92p67ho OR “tetra arsenic hexaoxide” OR “tetra arsenic oxide” OR “tetra‐arsenic hexaoxide” OR “tetra‐arsenic oxide” OR “tetraarsenic hexaoxide” OR “tetraarsenic oxide” OR trisenox OR trixenox)

AND

(13497‐05‐7 OR 22232‐80‐0 OR 302‐79‐4 OR 5688utc01r OR 75980‐27‐7 OR “retinoic acid” OR “vitamin a acid” OR tretinoin OR tretinoin OR ATRA OR “retinoic acid” OR “retin a” OR retin‐a OR vesanoid OR “all trans retinoic acid” OR “all‐trans‐retinoic acid” OR “beta all trans retinoic acid” OR “beta‐all‐trans‐retinoic acid” OR “trans retinoic acid” OR “trans‐retinoic acid”)

AND

(“clinical trial” OR “intervention study” OR “intervention studies” OR RCT OR “randomized controlled clinical trial” OR “randomized controlled clinical trial” OR “randomized clinical trial” OR “randomized clinical trial”)

Results limited to published articles in English, no publication date or geographical restriction. Literature search performed February 11, 2023.

## APPENDIX C FULL QUALITY ASSESSMENT OF INCLUDED STUDIES

### C.1

**Table:**

	APL2000	Aljurf	APML3	APML4	Lo‐Coco	SAL‐AIDA2000	AML17	APL0406	APL2006	Kayser	APL2012	APL15
Purpose stated clearly	1	1	1	1	1	1	1	1	1	1	1	1
Relevant literature reviewed	1	1	1	1	1	1	1	1	1	1	1	1
Design appropriate to question	1	1	1	1	1	1	1	1	1	1	1	1
No biases present	0	0	0	0	0	0	0	0	0	0	0	0
Sample described in detail	1	1	1	1	1	1	1	1	1	1	1	1
Sample size justified	0	0	0	0	1	0	1	1	0	0	1	1
Ethics approval reported	1	0	1	1	1	1	1	1	1	1	1	1
Informed consent gained	1	1	1	1	1	1	1	1	1	0	1	1
Outcome measures reliable	1	1	1	1	1	1	1	1	1	1	1	1
Outcome measures valid	1	1	1	1	1	1	1	1	1	1	1	1
Intervention described in detail	1	1	1	1	1	1	1	1	1	1	1	1
Contamination avoided	1	1	1	1	1	1	1	1	1	1	1	1
Cointervention avoided	1	1	1	1	1	1	1	1	1	1	1	1
Statistical reporting of results	1	1	1	1	1	1	1	1	1	1	1	1
Appropriate statistical analysis	1	1	1	1	1	1	1	1	1	1	1	1
Clinical importance reported	1	1	1	1	1	1	1	1	1	1	1	1
Drop‐outs reported	1	1	1	1	1	1	1	1	1	0	1	1
Appropriate conclusions	1	1	1	1	1	1	1	1	1	1	1	1
Clinical implications reported	1	1	1	1	1	1	1	1	1	1	1	1
Study limitations acknowledged	0	1	1	1	1	1	0	0	0	1	1	0
Total	17	17	18	18	19	18	18	18	17	16	19	18
Quality	Very good	Very good	Very good	Very good	Excellent	Very good	Very good	Very good	Very good	Very good	Excellent	Very good
